# Editorial

**Published:** 1862-01

**Authors:** 


### Editorial.

Local Medical Societies.—We announced, two or three months since, that the Chicago Medical Society proposed holding its regular meetings once a week through the winter, and commended the project. We are happy to state that the result, thus far, has been more favorable than we anticipated. The meetings have been held regularly, and with very few exceptions, well attended. They have been occupied in the hearing of papers, the reports of cases of disease, and the discussion of questions of direct practical interest. Such meetings are not only pleasant and profitable in themselves, but they exert a powerful influence in stimulating those who attend to constant habits of study and observation, that they may the more creditably sustain their part in the discussions and other exercises of each meeting. The Chicago Medical Society has been in active and efficient operation eight or ten years, and we hope it has before it a still more prosperous future.

Prof. Titus Deville.—We learn by a recent circular of the Manchester Royal School of Medicine and Surgery, of England, that our highly esteemed friend and the Emeritus Professor of Anatomy in the Medical Department of Lind University, Dr. Titus Deville, has been appointed to the chair of Practical Anatomy, for the Winter term, and to that of Surgical Anatomy, for the Summer term, in that old and well established institution.

We take great pleasure in noting this evidence of the appreciation of his merits; for as a teacher of Anatomy he certainly has few equals, either in this country or in Europe. We can assure such American physicians and students as intend visiting England, that they will meet a cordial reception at the Manchester Royal School, so long as Dr. DEVILLE remains a member of its faculty.

RUSH MEDICAL COLLEGE COMMENCEMENT.—The Annual Commencement of the Rush Medical College of this city, took place on the evening of Wednesday, Feb. 5th, 1862.

The exercises took place in the Lecture room of the College, and in the presence of a respectable audience.

Prof. D. BRAINARD, President of the Faculty, being absent, the degree of Doctor of Medicine was conferred on the following candidates by Prof. J. V. Z. BLANEY, who accompanied the ceremony by a brief and appropriate charge to the recipients, viz:

| | |
|---|---|
| Albert A. Ames, of Minnesota. | Clark E. Loomis, of Illinois. |
| Charles E. Allen, of Illinois. | J. Meek Lanning, of Iowa. |
| Stephen G. Armstrong, of Indiana. | W. Meacher, of Wisconsin. |
| Aurelius T. Bartlett, of Illinois. | F. R. Millard, do |
| Leonard L. Bennett, do | George J. Monroe, of Illinois. |
| George W. Beggs. do | Wm. McKnight, do |
| James Brown, do | Wm. Rush Patton, do |
| Elijah W. Boyles, do | H. W. Richardson, do |
| W. L. Cuthbert, do | Charles M. Richmond, of Indiana. |
| W. D. Carter, do | W. R. Russell, of Wisconsin. |
| J. Griffin Conley, of Wisconsin. | R. E. Stevenson, of Illinois. |
| Samuel M. Dunn, of Iowa. | S. B. Ten Broeck, do |
| Thomas G. Drake of Indiana. | J. Allen Torry, of Wisconsin. |
| James B. Farrington, or Tennessee. | A. H. Whipple, do |
| A. Z. Huggins, of Iowa. | D. B. Wren, of Ohio. |
| Jacob H. Houser, of Indiana. | J. A. Ward, of Illinois. |
| Riley B. Hayden of Michigan. | E. H. Winston, of Wisconsin. |
| Jacob M. Hagery, of Illinois. | |

The honorary degree of Doctor of Medicine, was also conferred on Dr. J. C. Taggart, of Beloit, Wisconsin.

The valedictory address was delivered by Prof. J. W. FREER, and was listened to with attention and profit.

Thus passed the Nineteenth Annual Commencement of this Institution.

Medical Department of Lind University.—The Third Annual Commencement of this School will be held on the evening of Tuesday, March 4th, 1862. The Valedictory Address will be delivered by Prof. J. H. Hollister. A premium will be awarded to the author of the best thesis; and the degrees will be conferred by the President of the Faculty, Prof. H. A. Johnson. The occasion will be one of interest, intellectually and socially; and the friends of the institution are cordially invited to attend.

Received.—From Blanchard & Lea, Philadelphia, the second edition of Gross' *Surgery*—for notice in our next; from Ticknor & Fields, Boston, *Religio Medici*, *Urn Burial*, and other papers, the writings of Sir Thomas Browne, M. D. —Also *Border Lines*, O. W. Holmes, M. D.'s introductory lecture,—both for future notice.

Any of our subscribers, or any of the former Secretaries of the Society, who may have copies of the *Transactions of the Illinois State Medical Society*, for the years 1853 and 1855, and who can spare the same, would confer a favor upon the editor of the Examiner, by forwarding them, by mail or express, at our expense.

Apology.—We hope our readers will excuse the late appearance of this and the preceding number of the Examiner. —The evil shall soon be corrected.

---

Chlorate of Potash as a Remedy for Fœtid Breath. —Many persons complain of fœtid breath who cannot attribute it to bad teeth or neglect to keep them clean; the gums and mucous membrane of the mouth are perfectly healthy. The bad odor must come either from the lungs or the stomach, and nine times out of ten it comes from the latter. In this case we have a simple, prompt and certain remedy in the chlorate of potash. Take, three hours after eating, a teaspoonful of a solution of six grammes of the chlorate in a hundred of sweetened water, and at the same time rinse the mouth with the solution.—*Boston Med. and Surg. Journal.*

Syphilo-Vaccination.—Dr. A. Viennois of Paris, has recently published a paper in the *Archives of Medicine* on the transmission of syphilis by Vaccination, illustrated by numerous cases of syphilis communicated simultaneously with vaccine lymph. The conditions of this transmissibility he believes to be the taking of blood with the lymph from a person affected with constitutional syphilis. And such matter, Viennois writes, will communicate *both* diseases; the vaccine vesicle appearing first. But after the usual period of incubation, the syphilitic tubercle also appears on the vaccinated part, and is soon after followed by secondary symptoms.—*Vaccination*, by A. N. Bell, M. D.

It is stated that Dr. Lavau, of Birac, called the attention of the Academy of Medicine of Paris to the importance of sulphuret of lime in the regeneration of bony substance. M. Lavau observed, more than twenty years ago, that this agent, diluted in olive oil and used in frictions to destroy itch, induces enlargement of the joints of the fingers. This remark led him to prescribe frictions with sulphuret of lime on the head of rickety subjects whose fontanelles were excessively large, or persisted beyond the normal period, and he obtained with surprising rapidity the ossification and obliteration of these membranous apertures. M. Lavau therefore surmises that the same treatment may perhaps be also applicable to the secretion of the periosteum in the great process of the reproduction of bone.—*Championniere's Journal of Pract. Med. and Surg.*

Leucorrhœa in Pregnant Women.—With a view of testing the accuracy of Cazeaux's assertion, that seven-eighths of all pregnant women had ulceration of the neck of the womb, Charrière examined one hundred pregnant women indiscriminately, as they offered themselves to his notice, and came to the following conclusions: Leucorrhœa precedes and gives rise to the ulceration of the cervix. The congested conditions and processes of hypertrophy taking place in the pelvic organs are the causes of this leucorrhœa. At first a physiological condition, it may become morbid under the influence of a bad state of health. Nearly two-thirds of pregnant women have leucorrhœa. Nearly eight-tenths of these have ulcers of the cervix. Treatment: Attention to the general condition, mild aperients and preparations of iron, and remedying disorders of digestive organs. Local treatment would doubtless frequently induce abortion.—*Bull. de Ther.* through *Lancet and Observer.*

New Method of Administering Chloroform.—At a recent meeting of the Obstetrical Society, Dr. Simpson described a plan of administering chloroform which he has now adopted in preference to that at present in use here. The present mode is to fold up a handkerchief and pour into the hollow a quantity of chloroform, and then hold it at some distance from the face, so as to admit of atmospheric air being inhaled along with the vapor. The new plan is to lay a single layer of handkerchief over the face, and let the chloroform fall on it drop by drop. The advantages are these: 1. That there is less danger to the patient from the smaller quantity applied at a time. 2. That anæsthesia is more speedily produced. 3. That the quantity of chloroform required is less. Various gentlemen who had made trial of the plan confirmed the value of this process; and Dr. Young in particular stated that he had kept a patient narcotized for ten hours with two ounces and a half of chloroform.—*British Med. Journ.*

Phthisis.—In a recent communication to the French Academy of Medicine, by M. Piorry, on the treatment of phthisis, he presents the following summary of conclusions:—

1st. Pulmonary phthisis is a collection of numerous morbid phenomena, and not a morbid unit.

2d. There does not exist, nor can there be, a special remedy for it, to destroy a unit which has no existence.

3d. That consequently iodine, tincture of iodine, no more than chlorine, salt, tar, can be considered as anti-phthisical.

4th. That it is necessary, in order to the proper treatment of phthisical persons, to appreciate and specify the particular organic affections which they present, and to meet them with appropriate remedies.

5th. That hygienic precautions, intelligently advised, may prevent the development of tubercle.

6th. That by proceeding in this way, by combating the particular affections which occur together or succeed each other, we have a rational treatment of phthisis, which can show a fair number of perfect cures, and a very large number of palliated cases.

Reproduction of the Inferior Maxillary Bone.—The May (1861), number of Champonniere's *Journ.* contains further interesting reports of successful operations for the reproduction of bone:—

M. Maissonneuve laid before the Academy of Sciences another instance of the reproduction of bone. In this case, the right side of the inferior maxillary was extracted *in toto*, with the articular condyle, and has been so perfectly replaced by the efforts of nature, that it is now almost impossible to discover which side of the jaw was removed by the surgeon. The patient was aged 35, a circumstance which imparts additional interest to the case, inasmuch as the generation of bone, at that period of life, is less active than in youth. Another singular detail is the preservation of the teeth, which were left by the operator attached to the gums, as moveable as a string of beads, and became subsequently consolidated, by the closing up of the ossified layers, secreted by the periosteum. Unfortunately, after having performed their functions for a space of two years, they, one after another, dropped out; but the patient, nevertheless, manages to masticate his food and to speak distinctly.

In another case related to the Academy, of the reproduction of both tibia and fibula, it is interesting to notice that both malleoli were included in the part removed.—*Berkshire Med. and Surg. Journal.*

We shall, in an early number, present to the readers of the Examiner, the details of an analogous case to the above, which the class of the Lind Medical Department have had an excellent opportunity of observing, in Prof. Andrews' Surgical Cliniques at the *Mercy Hospital.*

Quinine in the Dropsy of Scarlatina.—Dr. Hamburger has given this drug in forty-seven cases, and in forty-four improvement took place at once or in a very few days; in three cases only was there no change either for better or worse. The effects observed were, a diminution of the febrile symptoms of the subacute period, increase of the urinary secretion, which became more clear, absorption of the effused fluid, even the resolution of abscesses already formed, return of appetite and strength. The urine, nevertheless, continued to be albuminous for some time, but this was no obstacle to the progress of convalescence.

According to the summary which Dr. H. gives of his observations, it is in the chronic form of scarlatinous dropsy that the action of quinine gives the best results, and is manifested with the greatest rapidity; in cases of this kind improvement commences almost immediately after the first doses. At the commencement, so long as the acute period continues, the employment of quinine may be deferred for a few days, unless the danger is imminent.

On many occasions, Dr. Hamburger has seen the condition of the patient remain the same for many days, or to be gradually growing worse, the urine becoming very dark, and the dropsy increasing. The quinine was then given boldly, and a happy result was the consequence. If a marked improvement is not manifested at the end of three or four days, the remedy must be dropped; but even in this case it should not be regarded as entirely useless, for it seems to act upon the specific character of the disease.

The dose to be given is from half a grain to two grains, twice a day, for children, and three or four grains for adults. During the use of the quinine the diet should be carefully watched, great care being taken to avoid over-tasking the very irritable alimentary canal by overloading it with food or drink. —*Gaz. des Hop.*, from the *Boston Med. and Surg. Jour.*

New Procedure for the Ligature of the Superficial Palmar Arch.—Dr. E. Bœkel, Fellow of the University of Strasburg, has recently published, in the local *Medical Gazette*, some new indications which may guide the operator in his search for this artery, and permit him to secure it without unnecessarily extensive incisions.

'Place the thumb,' says Dr. Bœkel, 'in the greatest possible abduction, and draw a line from its ulnar edge across the palm of the hand. In front of this, which may be denominated the guiding line, draw a second in a parallel direction, at a distance of a third of an inch nearer to the fingers, or more correctly at an equal distance between the first line and the middle cutaneous fold of the palm; this is the precise position of the superficial arch, and if the skin and palmar fascia are divided here, the artery will be at once exposed, and found reposing on a layer of fatty tissue which separates it from the nerves and tendons. No apprehension of wounding these need therefore be entertained.

It will perhaps be alleged that no fixed rules can apply to an artery so irregular as the palmar arch; but it must not be forgotten that the anomalies alluded to refer less to the exact situation of the vessel than to the dimensions of its supplying branches. I have performed the ligature above twenty times on the dead subject, guided by these rules, and have never once failed in alighting on the artery in the exact position described.

An accurate knowledge of this anatomical detail has another advantage quite as great as that of giving increased facility in finding the artery, viz., it supplies us with the means of avoiding it. Phlegmonous inflammation beneath the palmar fascia, at the same depth as the arch, frequently requires incision, which is never extended toward the wrist without a certain amount of hesitation. The indications I have mentioned will permit the surgeon to use the knife with more boldness, and at the same time with greater safety, and they have already done me good service for this purpose.—*Med. and Surgical Reporter.*

New Surgical Propositions.—The following new Surgical principles are offered for criticism by Prof. Cooper of the *San Francisco Medical Press:*

1st. That atmosphere, admitted into the joints or other tissues, is not a source of irritation or injury, except where it acts mechanically; as, when admitted into a vein, by producing asphyxia; into the thoracic cavity, by its pressure, producing collapse of the lungs, or when, by the long continued exposure of a large amount of surface of any of the internal organs, whose normal temperature is much above that of the atmosphere, it reduces it so as to produce a morbid action.

2d. That the division of entire ligaments about the joints is no impediment to their ultimate strength and mobility; but on the other hand, this operation will often greatly facilitate the cure, by enabling the surgeon to open the affected part fully, for the purpose of applying medicinal substances to the articular surfaces, when these are ulcerated or otherwise diseased.

3d. That the only true mode of treating ulcerations of bone, however slight, within the joint, is to lay it open freely, and apply remedial agents directly to the part affected.

4th. That opening the joints early, in case of matter burrowing in them, is far more imperiously demanded than the opening of the parts thus affected, and the operation produces no further pain or inconvenience to the patient, in any respect, than when performed on parts remote from the joints.

5th. That after opening a large joint, the knee, for instance, by an incision several inches long, the wound should be kept open by the introduction of lint, or other similar substance, until the parts within the articulation become healthy, and in all cases, it should be made to heal by granulation.

6th. That extensive wounds, opening freely the large joints, such as the knee, (even when lacerated, as by a saw, which must necessarily heal by granulation,) do not as often give rise to violent symptoms as very small wounds, such as are made by the corner of a hatchet, an adze, or a pen-knife, which heal on the outside by first intention.

7th. That there are no known limits beyond which a tendon will not or can not be reproduced after division, provided the parts are made to heal by granulation, and that the present acknowledged rule of two inches being the maximum distance in which the divided ends of a ligament, or tendon can safely be separated, has not the least foundation in fact.

Clergyman's Sore Throat.—A writer in a London secular journal, suggests the following, as an explanation of the cause of this dread of public speakers, more especially of the pulpit:

If we look at the creatures who use their throats for vocal purposes—singing birds for example—it will be found that in doing so they always lift up their heads. The canary raises its head before it begins to sing, so that every muscle of the throat is brought into full play. The dog, when he barks, follows the same rule and for the same reason.

To continue the analogy. We find all public speakers, except the clergy, adopting—not from instinct, but from necessity, happily for them—the same law. Members of Parliament, barristers and actors, all occupy a different position towards their audience from that of clergymen. The barrister stands below, and speaks up to the judge and jury, thus allowing full scope for the expansion of the muscles of the throat and chest. The actor likewise addresses an audience arranged in tiers above him, so that he can speak in an easy and natural manner. But we, clergy, adopt just the reverse of all this. Placed in a pulpit inconveniently elevated, we have to speak down to the people. The muscles of the throat are necessarily compressed. Congestion takes place, perhaps only a little each time, until the power of littles combines to produce that relaxed condition of the throat which completely disables the clergyman, and renders him unfit for public speaking.

Many clergymen have told me that they would prefer to preach two sermons than read the prayers once, so far as a trial of their voice is concerned, and the reason is obvious, if the analogy I suggest hold good.

Having suffered myself, I can speak freely on the subject; and after trying many remedies, without success, I at last regained my former strength of voice by adopting the following plan: —

I learned the prayers by heart. This enables a clergyman to speak without stooping, while it certainly adds to the solemnity of the prayer. The next step was to preach without the manuscript, or at least to know the sermon so well that it is not necessary to read with the head downwards. The preacher thus can address himself to the audience with perfect ease to himself, owing to the unrestricted action of the vocal organs.

In my own case, relief was very soon given to the congested vessels, and gradually they became quite restored, and for some years they have continued so, whereas, before this plan was adopted, one sermon a day produced hoarseness.

This is good physiology, and we commend the suggestion to the attention of the profession.

Position in Etherization.—Dr. C. T. Jackson, in a letter to the *Boston Med. and Surg. Journal*, communicates some additional facts relative to the influence of the position of animals during etherization, a point strongly urged, now, by all writers on the use of anæsthetics.

Dr. J. says "I had occasion to converse with a very intelligent and skilful veterinary surgeon, who resides near Boston (Dr. A. B. Wilton, of Dorchester,) from whom I obtained the results of his extensive experience in operating upon domestic animals, under the influence of ether.

He informed me that if a horse is etherized and laid on his back, with his nose up, he will certainly die, owing to the falling back of the tongue, and the consequent pressing down of the epiglottis, so as to produce suffocation. He mentioned four instances, in which he had witnessed the death of horses, during the operation of castration, from the effects of ether and the above-named position. His experience with ether had also proved that death from it never takes place if the horse is laid on his side, with his nose horizontal. His experience had also been extended to the etherization of cows, particularly in cases where mechanical aid was required in removal of the calf, and he has never lost one of them from the effects of ether. When he has had occasion to kill useless horses or dogs, he has made use of ether, aided by the position named, and he says they die easily, without a struggle—asphyxia resulting from closing of the glottis during the anæsthetic state. These facts corroborate those observed on the human subject by Dr. Petrie,of Liverpool.—See *Braithwaite's Retrospect*, Part xliii., p. 275.

